# Transosseous suture *in situ* repair treatment of a femoral anterior cruciate ligament avulsion fracture in a 30-year-old male patient: a case report and review of the literature

**DOI:** 10.3389/fsurg.2025.1598881

**Published:** 2025-07-10

**Authors:** Yifei Liufu, Muyuan Hou, Fangjun Xiao, Jiangfeng Lv, Junxing Yang

**Affiliations:** Department of Orthopedics, Shenzhen Hospital (Futian) of Guangzhou University of Chinese Medicine, Shenzhen, Guangdong, China

**Keywords:** anterior cruciate ligament, femoral-side, avulsion fracture, transosseous suture *in situ*, case report

## Abstract

**Background:**

Avulsion fractures typically occur at the tibial insertion site of the anterior cruciate ligament (ACL), while femoral ACL injuries usually involve midsubstance tears rather than bony avulsions. In previous case reports, femoral end avulsion fractures have been reported more often in skeletally immature patients.

**Case presentation:**

In this case report, we present a 30-year-old male patient who presented with a femoral-sided ACL avulsion fracture that was treated arthroscopically using a transosseous suture *in situ* repair technique. The avulsed fragment was surgically stitched back to its normal anatomical position. After the surgery, the patient followed a standard ACL rehabilitation program. Three-month postoperative MRI confirmed good ACL morphology. At the 1-year follow-up, the patient showed no signs of pain, motion limitation, or instability. Physical examination revealed an intact ACL, with normal results on axial shift and Lachman tests, indicating the effectiveness of the procedure.

**Conclusion:**

The transosseous suture *in situ* repair technique is an effective and precise treatment for ACL avulsion fractures. Its application should be considered based on the location of ligament injury and the integrity of the fragment.

## Background

Anterior cruciate ligament (ACL) avulsion fractures rarely occur at the femoral end in ACL avulsion injuries. This is because the femoral attachment site has stronger bone density and trabecular structure, which resists avulsion forces. Conversely, the tibial insertion (e.g., tibial eminence) is biomechanically weaker and more vulnerable to tensile forces during knee hyperextension or rotational trauma, making tibial avulsions more common. Femoral ACL injuries typically involve midsubstance ligament tears rather than bony avulsions. This phenomenon is particularly pronounced in adolescents, whose unclosed epiphyses further reduce the tensile strength of the bone (1). Among previously reported cases, only seven involved adult patients (2–6). The optimal surgical approach for treating anterior cruciate ligament avulsion injuries remains inconclusive, with options including early repair or delayed reconstruction. In this case report, we present a 30-year-old male patient with a femoral-sided ACL avulsion, treated using a transosseous suture *in situ* repair technique, resulting in a satisfactory outcome.

## Case presentation

**Figure 1:**
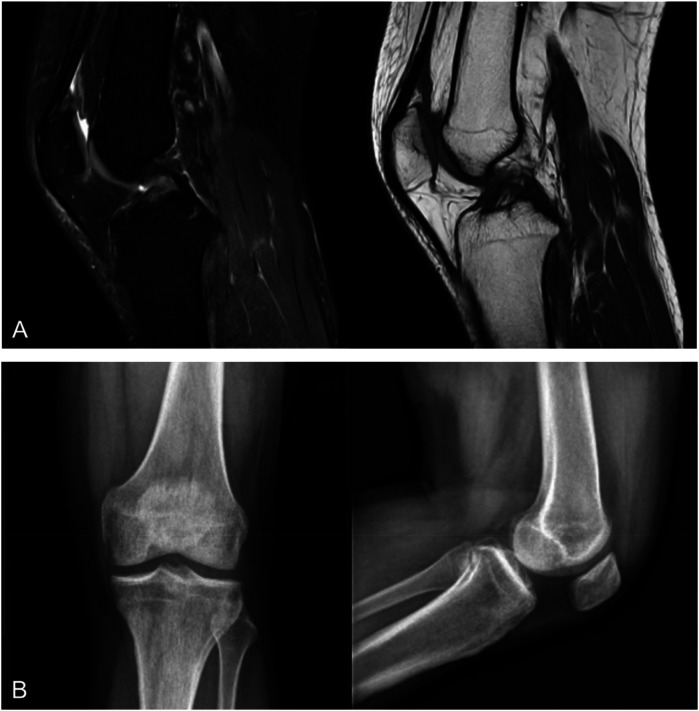
**(A)** MRI scans of the left knee showing an anterior cruciate ligament tear; however, the avulsed fracture fragment was not clearly visualized; **(B)** radiographs also failed to clearly show the avulsed fracture fragment.

A 30-year-old male patient presented to the hospital with sharp pain and limited movement in his left knee, persisting for 7 days following an accidental sprain while playing basketball. On physical examination, there was no obvious swelling of the left knee. The ROM of the left knee was 0°–70°. The anterior drawer test, Lachman test, and pivot shift test were positive, while the posterior drawer test was negative. MRI revealed a tear of the anterior cruciate ligament in the left knee, along with a posterior cruciate ligament injury and a mild injury to the patellar ligament. Radiological examination did not clearly show an avulsed fracture fragment (Figure 1). Based on these findings, we decided to proceed with surgical fixation of the fracture fragment.

Intraoperatively, the femoral attachment of the ACL was confirmed to be avulsed, the femoral-sided bone fragment was fully connected to the ACL, the intrasubstance of the ACL remained intact, and there was no obvious damage to the medial and lateral menisci, posterior cruciate ligament, articular cartilage, or other structures. Because the fracture occurred between bones, this type of fresh fracture often demonstrates strong healing potential. The *in situ* bone suturing technique offers advantages such as reduced trauma and a shorter recovery period. In contrast, anterior cruciate ligament reconstruction would result in higher medical costs and greater tissue damage. Therefore, we decided to repair the fracture using the transosseous suture *in situ* repair technique.

**Figure 2:**
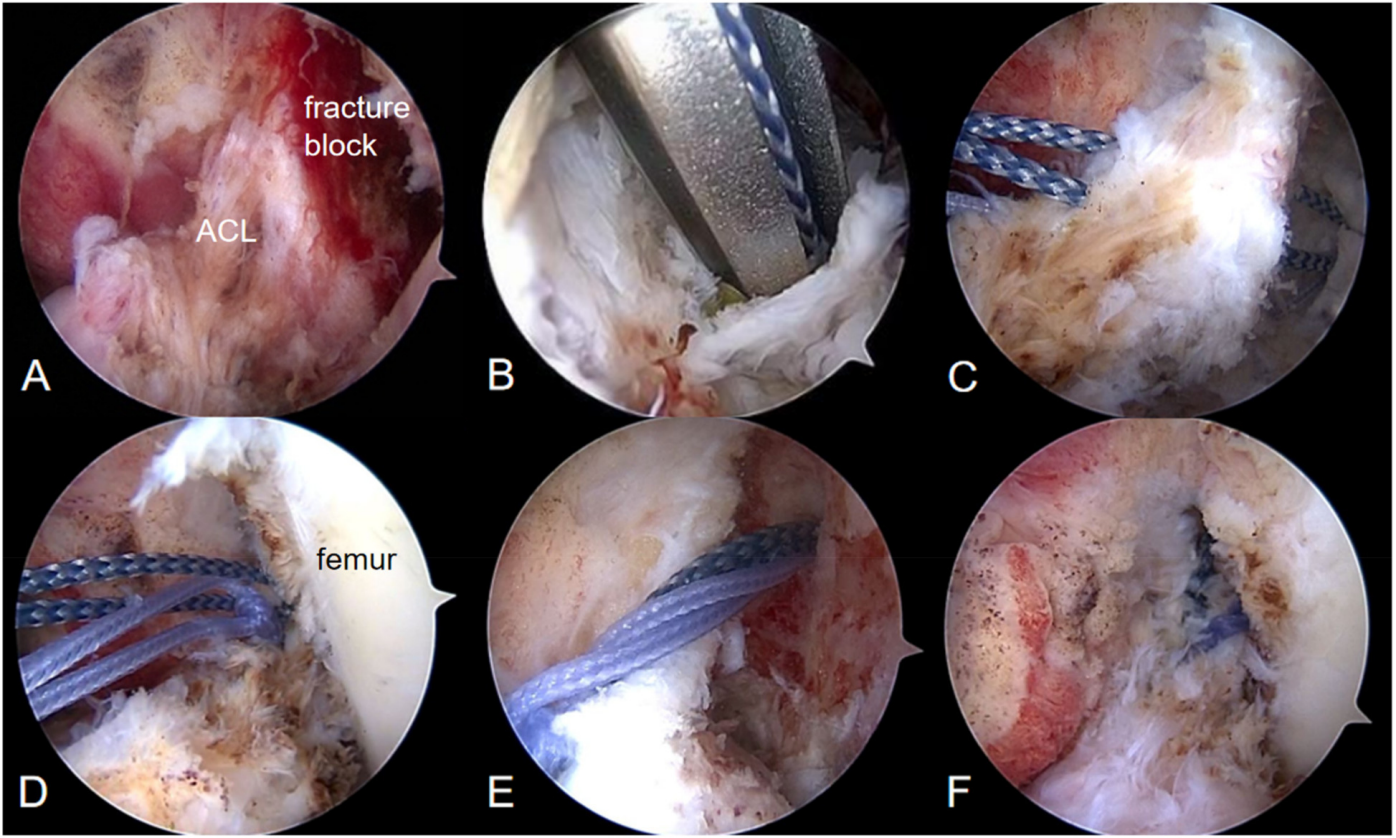
**(A)** The ACL is intact and the fractured fragment is fully connected to the ACL; **(B)** a metal bone anchor with suture is inserted *in situ* at the side of the femoral avulsion; **(C,D)** the suture is passed through the bone fragments and part of the ACL, then knotted and fixed; **(E)** bone tunnels are drilled into the femoral epicondyle; and **(F)** the avulsed fragment was stitched back to its normal anatomical position.

Under arthroscopic visualization, a metal bone anchor with suture was inserted at the origin of the anterior cruciate ligament tear *in situ*. Using a threading technique, the suture was passed through the bone fragments and part of the anterior cruciate ligament, then tied and secured in place. Afterward, two bone tunnels were drilled into the lateral femoral condyle using Kirschner wires. The ends of the tied suture were then threaded out of these bone tunnels and tied on the lateral side of the femur. During surgery, the origin of the anterior cruciate ligament was found to be securely fixed (Figure 2). The sutured metal bone anchor used in the procedure was supplied by Johnson & Johnson.

Intraoperative exploration confirmed that the femoral attachment of the ACL was firmly fixed, and the Lachman test was negative.

**Figure 3:**
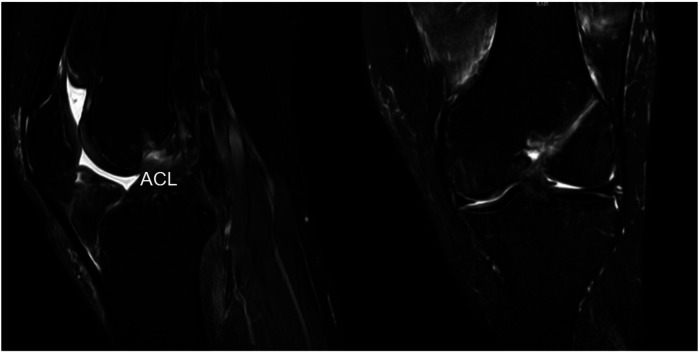
MRI scans of the sagittal and coronal planes showing good morphology after fixation.

After the surgery, the patient followed a standard ACL rehabilitation program. From immediately after surgery through the first week, he was advised to raise his leg on a pillow with the toes pointing upward and a space under the knee to keep it straight. He was instructed to perform intermittent isometric contractions of the quadriceps, which involve keeping the knee straight, contracting the thigh muscles, holding the contractions for a few seconds, then relaxing, and repeating the exercise. From weeks 2–4, he began passive flexion and extension exercises of the knee joint, gradually increasing the angle from 0° to 90°. He also continued isometric quadriceps strength training. From weeks 5–8, the range of flexion and extension angles of the knee joint was gradually increased to achieve a full range of motion, and additional exercises such as inversion and eversion were introduced to fully restore joint mobility. By 3 months, the patient was able to walk without a brace. MRI at that time showed good ACL morphology following surgical fixation (Figure 3).

At the 1-year follow-up, the patient showed no signs of pain, motion limitation, or instability. His Lysholm score was 93, HSS score was 82, and IKDC score was 88. Physical examination revealed an intact ACL, with normal results on axial shift and Lachman tests, indicating the effectiveness of the procedure.

## Discussion

Femoral-sided ACL avulsion fractures are clinically very rare. These injuries mainly occur when the tension of the ACL in the arc of motion of the knee is increased by the force of the internal rotation of the tibia. If additional force is then applied to the posterior surface of the tibia in a forward direction, it may lead to ACL injuries. This mechanism is especially relevant in individuals with an immature skeleton, where such injuries can lead to an avulsion fracture at the attachment of the ACL (1). However, rotational movements involving knee valgus and external tibial rotation have also been reported as potential mechanisms for such injuries (7).

ACL avulsion fractures are uncommon in adults and often require surgical intervention, particularly in cases involving displaced bony fragments or significant functional impairment. Various surgical techniques have been described, including pullout suture (PS) repair, arthroscopic anchor fixation, and open reduction with internal fixation, each offering distinct advantages and limitations. The PS technique is widely used, especially in cases of comminuted avulsion, due to its adaptability and the ability to preserve native ligament tissue. For example, Tharakulphan et al. (5) reported a successful PS repair in a 32-year-old male patient who returned to full preinjury activity levels within 5 months. Similarly, Hasegawa et al. (6) employed an arthroscopic double-bundle pullout technique to treat a femoral-sided avulsion involving both ACL bundles, and the patient was able to resume sports without any signs of instability at the 1-year follow-up. Screw fixation, whether performed via open or arthroscopic approaches, offers robust mechanical stability. Zabierek et al. (4) and Shah et al. (3) reported favorable outcomes using screw fixation; however, rehabilitation was longer due to the more invasive nature of the procedures and the complexity of associated injuries. Collectively, these cases highlight the importance of individualized treatment selection, considering factors such as patient age, activity level, fracture morphology, and the presence of associated injuries. In our case, the ACL was completely intact and firmly attached to the avulsed bone fragment. We adopted a transosseous suture *in situ* fixation approach, which offers multiple advantages: it preserves the proprioceptive and vascular characteristics of the native ligament, enables anatomical bone-to-bone healing, and minimizes surgical trauma. The recovery trajectory and the final outcome of the patient were consistent.

The treatment of femoral-sided ACL avulsion injuries should consider anatomical repositioning and preservation of the native ACL (4). Literature on femoral-end ACL injuries suggests that the choice of repair technique depends largely on the location of the ligament injury and the integrity of the stump. According to the modified Sherman classification, type I injuries are characterized by high stump integrity and good stump quality, making them appropriate for repair using anchor nails (8). Another study showed that early proximal ACL tears can also be repaired using anchor nails to obtain knee stability with good clinical outcomes. Studies have shown that the use of stump repair technique in the early stage of femoral-end ACL injuries can restore knee stability, maximize preservation of proprioception and the normal fibrous structure at the tibial attachment, protect the articular cartilage, reduce the complications associated with ligament reconstruction, shorten postoperative recovery time, and preserve bone volume for revision surgery. Histological studies have found that ACL stumps contain a certain number of neuroproprioceptors and blood vessels. The neuroproprioceptors facilitate the recovery of postoperative motor function, while the blood vessels play an important role in promoting tendon–to-bone healing. Mukhopadhyay et al. (9) investigated 13 cases of acute type I femoral-sided ACL avulsion injuries (all treated with arthroscopic pullout suture repair), with a mean follow-up of 31.3 months, and observed that ACL repair in these patients yielded good short-term outcomes. Experimental studies by O'Donoghue and others (10) have demonstrated that the avulsed stump starts to resorb as early as 2 weeks postinjury, which affects the outcome of the repair; therefore, performing the repair in the acute stage is imperative. In our patient, the decisions and preparations for ACL repair were aggressive and prompt, with the goals of achieving anatomical repositioning, stabilizing the bone mass, and facilitating healing of the ACL. Owens et al. (11) studied ACL repair using the transosseous suture technique and reported good results. Kohl et al. (12) also supported the effectiveness of one-stage *in situ* suture repair for ACL attachment-point avulsion injuries, demonstrating good results. In this case, we used the transosseous suture *in situ* repair technique to repair a simple femoral-sided ACL avulsion fracture; this approach preserved the natural ACL stump, did not require the creation of a tibial tunnel, and promoted stronger bone-to-bone healing than tendon-to-bone healing. In addition, this technique does not require a skin incision over the distal femur for fixation, nor does it require a second surgery to remove the fixation device, resulting in good cosmetic outcomes at a lower cost. However, compared with ACL reconstruction, transosseous suture *in situ* repair has some limitations: for example, it is mainly applicable to avulsion fractures at the ACL attachment but not to ligament parenchyma injuries. Mariani et al. (13) compared the outcomes of ACL repair and reconstruction and found that postoperative stability and mobility of the joints were inferior in the *in situ* suture repair group compared to the reconstruction group; based on these results, they concluded that reconstruction is preferable for ACL injuries. However, it is important to note that all samples in their study involved parenchymal cruciate ligament injuries, and the sample size was small, which discredits the applicability of the *in situ* suture repair technique to ACL avulsion fractures. Therefore, we believe that transosseous suture *in situ* repair is the preferred approach for ACL attachment-point avulsion injuries, while ligament reconstruction is preferred for parenchymal ligament injuries. However, the number of studies in this area is limited, and we need a large number of well-designed controlled clinical trials to validate this conclusion.

## Conclusion

In conclusion, in the present case study, the transosseous suture *in situ* repair technique is a precise treatment for ACL avulsion fractures, which could assist in restoring knee stability while maximizing the preservation of proprioception and the normal fibrous structure on the femoral side. This technique should be considered based on the location of ligament injury and the integrity of the fragment.

## Data Availability

The original contributions presented in the study are included in the article/Supplementary Material, further inquiries can be directed to the corresponding author.
